# Rhodamine B Doped ZnO Monodisperse Microcapsules: Droplet-Based Synthesis, Dynamics and Self-Organization of ZnO Nanoparticles and Dye Molecules

**DOI:** 10.3390/nano10122351

**Published:** 2020-11-27

**Authors:** Najla Ghifari, Bertrand Cinquin, Adil Chahboun, Abdel I. El Abed

**Affiliations:** 1 Laboratoire Lumière Matière et Interfaces (LuMIn), Institut d’Alembert, Ecole Normale Supérieure Paris Saclay, CentraleSupélec, CNRS, Université Paris-Saclay, 61 avenue du Président Wilson, 94235 Cachan, France; najla.ghifari@ens-paris-saclay.fr; 2 Laboratoire des Couches Minces et Nanomatériaux (CMN), FST Tanger, Université Abdelmalek Essaadi, Tangier 90040, Morocco; adchahboun@uae.ac.ma; 3 Institut Pierre-Gilles de Gennes, IPGG, UMS 3750, 6 rue Jean Calvin, 75005 Paris, France; bertrand.cinquin@espci.fr

**Keywords:** ZnO nanocrystals, microcapsules, droplet microfluidics, colloidal aggregation, meso-crystallisation

## Abstract

In the present work, droplet-based microfluidics and sol-gel techniques were combined to synthesize highly monodisperse zinc oxide (ZnO) microspheres, which can be doped easily and precisely with dyes, such as rhodamine B (RhB), and whose size can be finely tuned in the 10–30 μm range. The as-synthesized microparticles were analyzed by scanning electron microscopy (SEM), transmission electron microscopy (TEM), and confocal microscopy. The results reveal that the microspheres exhibit an excellent size monodispersity, hollow feature, and a porous shell with a thickness of about 0.6 μm, in good agreement with our calculations. We show in particular by means of fluorescence recovery after photobleaching (FRAP) analysis that the electric charges carried by ZnO nanoparticles primary units play a crucial role not just in the formation and structure of the synthesized ZnO microcapsules, but also in the confinement of dye molecules inside the microcapsules despite a demonstrated porosity of their shell in regards to the solvent (oil). Our results enable also the measurement of the diffusion coefficient of RhB molecules inside the microcapsules ($D_{RhB}=3.8\times 10^{-8}$ cm${}^{2}$/s), which is found two order of magnitude smaller than the literature value. We attribute such feature to a strong interaction between dye molecules and the electrical charges carried by ZnO nanoparticles. These results are important for potential applications in micro-thermometry (as shown recently in our previous study), photovoltaics, or photonics such as whispering gallery mode resonances.

## 1. Introduction

Zinc oxide (ZnO) is a very promising semiconductor material. It is particularly appealing because of its outstanding optical and electrical properties [1]. In particular, it has a direct and wide bandgap of 3.37 eV in the near ultraviolet spectral region, making it a very interesting material for optoelectronic and photonic applications in the UV or blue spectral range [2]. It has attracted a lot of interest in many academic and technological domains such as electronics, optoelectronics, photocatalysis [3,4,5], and water treatment [5,6]. For energy applications [7], it has been shown that dye-doped ZnO nanoparticles are prone to improve the performance of photovoltaic cells, thanks to the so-called down-shifting [8] and down-converting [9] processes. In addition, since this semiconductor material exhibits a characteristic emission spectrum including UV emission for ZnO-based quantum photonics, it has promise as ZnO-based single-photon sources (SPSs), which is of great benefit in quantum information applications [10]. In particular, it has been demonstrated that the improvement of ZnO’s microstructures allows for the enhancement of its performance. Hence, ZnO microparticles exhibit tremendous potential for applications in zinc-nickel batteries [11], photocatalysis [12], gas sensors [13], and adsorptive removal of organic wastewater pollutants [14].

Various synthetic approaches using different chemical and physical methods [15], such as electro-deposition [16,17,18], sol-gel [19], and hydrothermal [14,20,21] have been employed to fabricate several types of ZnO nano- and microstructures including nanorods [16,22,23], nanowires [20,24], and micro- [14,21,25] and nano-spheres [26]. Nevertheless, the fabrication of well-defined micrometer-sized particles is still challenging. Among all the aforementioned studies, only two studies [21,25] are related to the synthesis of ZnO microparticles, which suffer from a poor control of the particle sizes and a narrow particle size distribution, because of the use of a standard hydrothermal condensation procedure. The fabrication of ZnO microspheres and microcapsules with a highly monodisperse and controlled size in the micrometer range (and above) is highly desirable for many applications such as a highly sensitive optical biosensing based on whispering gallery mode (WGM) detection [27].

Microfluidic methods have been applied previously to synthesize ZnO nanoparticles with a controlled size and shape [28,29], and various experimental parameters were shown to have a significant effect on the final properties of these nanoparticles. For instance, Zukas et al. investigated the effect of reagent concentrations, reactor design, temperature, and residence time on ZnO particle size, size distribution, and morphology by using a co-flow microfluidic geometry [6]. Baruah et al. reported the synthesis of ZnO nanospindles, nanospheres, and nanosheets with different sizes by changing the microfluidic channel dimensions and the flow rate [4]. A continuous synthesis of ZnO nanoparticles was reported by Kang et al. using a silicon-based microfluidic system and a time pulsed mixing method [30]. Ladanov et al. reported the synthesis of ZnO nanowires by hydrothermal growth on a silicon substrate [31]. However, all the above-mentioned microfluidic studies were mainly devoted to the synthesis of ZnO nanoparticles with sizes ranging at the sub-micron scale, and thus have some limitations, notably the precise control of the final particle size, size distribution, and hence for achieving the required performance features.

Recently, we reported a new approach combining droplet-based microfluidics and sol-gel techniques, which enables the synthesis of highly monodisperse ZnO hollow microspheres [32], as well as their application in high throughput fluorescence-based thermometry within optofluidic microsystems [33]. In the present work, we focus on the structural properties of the synthesized ZnO microspheres (microcapsules) and the organization of ZnO nanocrystals building blocks of their shell. We investigated the effect of different parameters, such as the effect of flow rates of the dispersed and the continuous phases on droplets size. We also investigated the structure of the shell of the microspheres at the microscale using scanning electron microscopy (SEM) and confocal microscopy in the presence of a charged dye (rhodamine B) and used transmission electron microscopy (TEM) to have better insight into the organization of the primary ZnO nanocrystals building blocks of the microcapsules. Our droplet-based microfluidics approach enables the production of well-defined monodisperse zinc oxide microcapsules with a high control of size and chemical composition according to the use of highly monodisperse droplets as soft-templates for their synthesis. Such a feature may be used efficiently in the future as whispering gallery modes (WGMs)-based optical micro-resonators for biosensing applications, owing to the well controlled spherical shape of the synthesized microparticles and the high refractive index of ZnO (e.g., 2), as demonstrated few years ago by Moirangthem et al. [27].

## 2. Experimental Section

The synthesis of colloidal ZnO nanoparticles (NPs) was performed using sol-gel. First, 0.6 g of zinc acetate dehydrate Zn(CH${}_{3}$COO)${}_{2}$:2H${}_{2}$O (99.999%, Sigma-Aldrich) was used as ZnO precursor and dissolved in 5 mL of methanol. Next, the solution was heated at 60 °C under magnetic stirring during 1 h, until the zinc acetate dehydrate was fully dissolved and the mixture turned into a homogeneous and transparent solution. These nanoparticles served as primary building units for ZnO droplets and particles. Afterwards, the resulting ZnO nanoparticles dispersion was directly transferred inside syringes and connected to the inlet of the microfluidic chip for droplets generation. All reagents were of analytical grade and used as received without any further purification.

The fabrication of the microfluidic device employed in our experiments, for the generation and collection of ZnO droplets, involved patterning channels in PDMS (polydimethylsiloxane) using conventional soft-lithography methods [34,35], mainly involving four steps:

(i) The fabrication of a master mold via photolithography. In this first step, a silicon wafer was coated with a layer of SU8-2025 photoresist (MicroChem Corporation (Newton, MA, USA)) by spin-coating through two cycles. For the work involved in this study, we used MJB4 mask aligner from SUSS MicroTec with an I-line type lithography optic system through an exposure wavelength of 365 nm. The mask was accurately aligned with the silicon wafer, then irradiated with UV light through the pattern on the photomask.
(ii) The previously fabricated master mold was used to produce a PDMS-based negative replica of the template. The PDMS was first mixed with curing agent with a weight ratio of 10:1, and the mixture was then degassed using a vacuum pump at room temperature.
(iii) The solution was poured onto the mold and placed in the oven for polymerization at 75 ${}^{\circ }$C for 2 h. The PDMS stamp was then removed from the mold, and a replica of the microchannels was obtained. Two inlets (to introduce the carrier oil and the dispersed phase into the channels) and one outlet (to collect droplets of ZnO nanoparticles dispersion in a Petri dish) ports were punched out using a Miltex biopsy punch with plunger of 1-mm diameter.
(iv) The PDMS stamp and the glass slide (50 × 75 mm) were eventually cleaned and dried (with compressed air), treated with oxygen plasma for 20 s to enable their bonding, and sealed to make the microfluidic device.

A commercial surface coating agent (fluorosilane) dried with N${}_{2}$ was used to increase the wettability of the oil phase on the channel walls of the microfluidic device. This device also contained a main (square) channel cross section of about 60 μm × 60 μm and an output for the collection of droplets in a Petri dish. 3M${}^{\mathrm{TM}}$ Novec${}^{\mathrm{TM}}$ HFE 7500${}^{\mathrm{TM}}$ fluorocarbon oil ((C${}_{3}$F${}_{7}$CF(OC${}_{2}$H${}_{5}$)CF(CF${}_{3}$)${}_{2}$), 3M) was used as the carrier phase. This oil has many advantages for our study. It has has a density of 1.61 g/cm${}^{3}$, which allows for ZnO droplets to spread at the air–oil interface and to form monolayers (or multilayers at the oil surface). It is also chemically a highly inert fluid, which does not cause PDMS swelling and does not solubilize most non-fluorinated organic molecules, including droplet contents. Droplets were stabilized using a commercial non ionic fluorinated polymer surfactant, namely dSURF${}^{\mathrm{TM}}$ (Fluigent).

**Figure 1:**
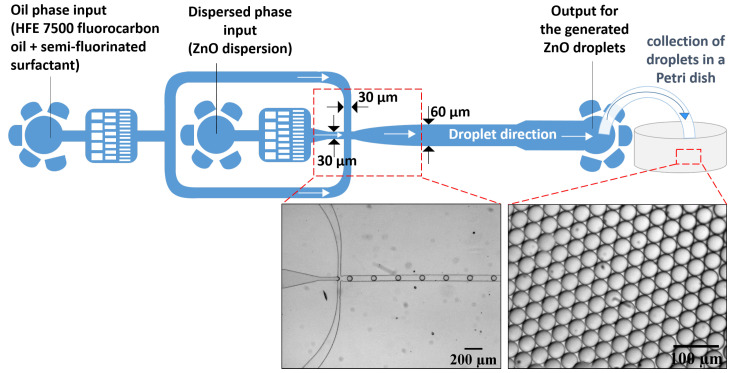
(**top**) Schematic illustration of the flow-focusing microfluidics design for zinc oxide droplet formation. (**bottom**) Optical micrographs of real-time generation of stable and monodisperse zinc oxide droplets.

The dispersed phase consisting of zinc acetate solution flowing into the central microfluidic channel undergoes external forces applied by the interfacial tension as well as the continuous phase flowing into two orthogonal micro-channels, resulting in the breakup of zinc acetate solution into small droplets (see Figure 1). The formation of droplets is achieved when the interfacial forces dominate the viscous forces, which is defined by the capillary number ($C_{a}=\frac{\mu U}{\sigma }<<1$, where μ is the viscosity, *U* is the velocity, and σ is the interfacial tension).

Once the droplets are generated, they are transported along the microfluidic channel by the carrier oil phase and moved towards the outlet. The droplets are then collected in a Petri dish (half-filled with a solution of HFE 7500), as shown in Figure 1, and kept at room temperature under atmospheric pressure during 48 h. The drying temperature used was 80 ${}^{\circ }$C for 5 h to obtain spherical zinc oxide microparticles. The used flow rates of the carrier oil ($Q_{c}$) and the dispersed phase (ZnO dispersion) for the generation of ZnO droplets ($Q_{d}$) were set using Nemesys syringe pumps (Cetoni GmbH).

## 3. Results and Discussion

### 3.1. Structural Properties of Synthesized ZnO Microparticles

**Figure 2:**
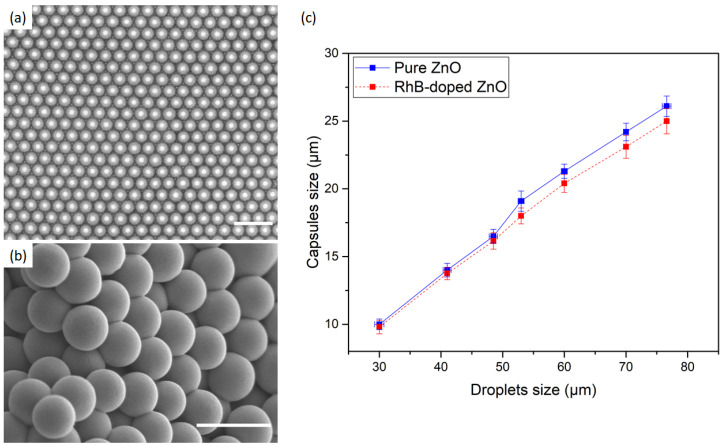
(**a**) Optical micrograph of ZnO microspheres before complete drying at 80 ${}^{\circ }$C and (**b**) SEM image of the corresponding fully dried microspheres. (**c**) Effect of droplets size on the final size of zinc oxide microcapsules. Scale bars: (**a**) 60 μm; and (**b**) 30 μm.

Figure 2a,b shows typical optical microscopy and SEM images of the synthesized ZnO microparticles, respectively. In this example, highly monodisperse microspheres with a diameter of 21.2 μm (±0.2 μm) were obtained from microdroplets with an initial diameter equal to 60 μm (±1 μm). The size of the microparticles is approximately one third of the size of the initial droplets. Figure 2c illustrates the observed linear dependence between the size of the microspheres and the size of the corresponding microdroplets with a linear coefficient of about 0.33 (which corresponds to a reduction factor of the droplet volume of about 3). In the case of pure ZnO, one should remark, however, the existence of a small shift in microspheres size of about +1.5μm for droplets size values above 50 μm, as can be seen in the blue curve of Figure 2c. Interestingly, such a shift is not observed when ZnO is doped with rhodamine B (red curve of Figure 2c, for which $\left[RhB\right]/\left[ZnO\right]≃2\times 10^{-4}$).

**Figure 3:**
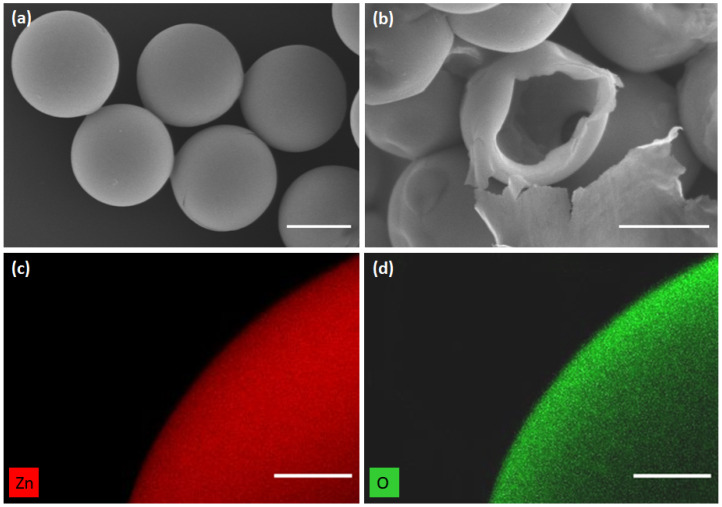
(**a**) SEM images of 16.5 μm size zinc oxide microcapsules; (**b**) shell thickness was found about 0.6 μm; and (**c**,**d**) element mapping images of the ZnO microspheres. Scale bars: (**a**,**b**) 10 μm; and (**c**,**d**) 2 μm.

Figure 3 depicts SEM images of ZnO microparticles obtained from droplets with a diameter of 48 ± 0.5 μm. It is observed that the microparticles have a spherical shape with a mean external diameter of about 16.5 ± 0.4 μm. Figure 3b shows clearly that such microspheres have a hollow structure with a mean shell thickness of about 0.6 ± 0.1 μm. Besides, EDS elemental mapping demonstrates that Zn and O are distributed uniformly in the microspheres (see Figure 3c,d). It is worth noting that the measured shell thickness is in good agreement with the calculated one as detailed hereafter. Indeed, assuming reasonably that the droplet content, i.e., ZnO nanoparticles dispersion, does not dissolve in the continuous fluorocarbon oil phase, the mass of ZnO ($m_{ZnO}$) of the overall ZnO nanocrystals contained in the droplet should be conserved in the microcapsule shell. Hence, $m_{ZnO}=CV_{drop}M_{ZnO}$, where *C*, $V_{drop}$, and $M_{ZnO}$ represent the concentration of ZnO precursor (zinc acetate), the volume of a single droplet, and the ZnO molar mass, respectively. Let $A=\pi D_{p}^{2}$ be the surface area of a microcapsule of a diameter $D_{p}$, *h* its shell thickness, and ρ its mass density. One may write then $m_{ZnO}=\rho \pi D_{p}^{2}h$ and deduce *h* according to the following equation:

(1) $$h=\frac{CV_{drop}M_{ZnO}}{\pi D_{p}^{2}\rho }$$

Considering the standard value of zinc oxide mass density, ρ = 5.6 g/cm${}^{3}$, the used concentration C=0.5M of ZnO precursor and the initial microdroplet size, $D_{drop}$ = 48 μm, one may deduce a value for the thickness of the microcapsule shell of about 0.6 μm, which is in very good agreement with the measured value.

**Figure 4:**
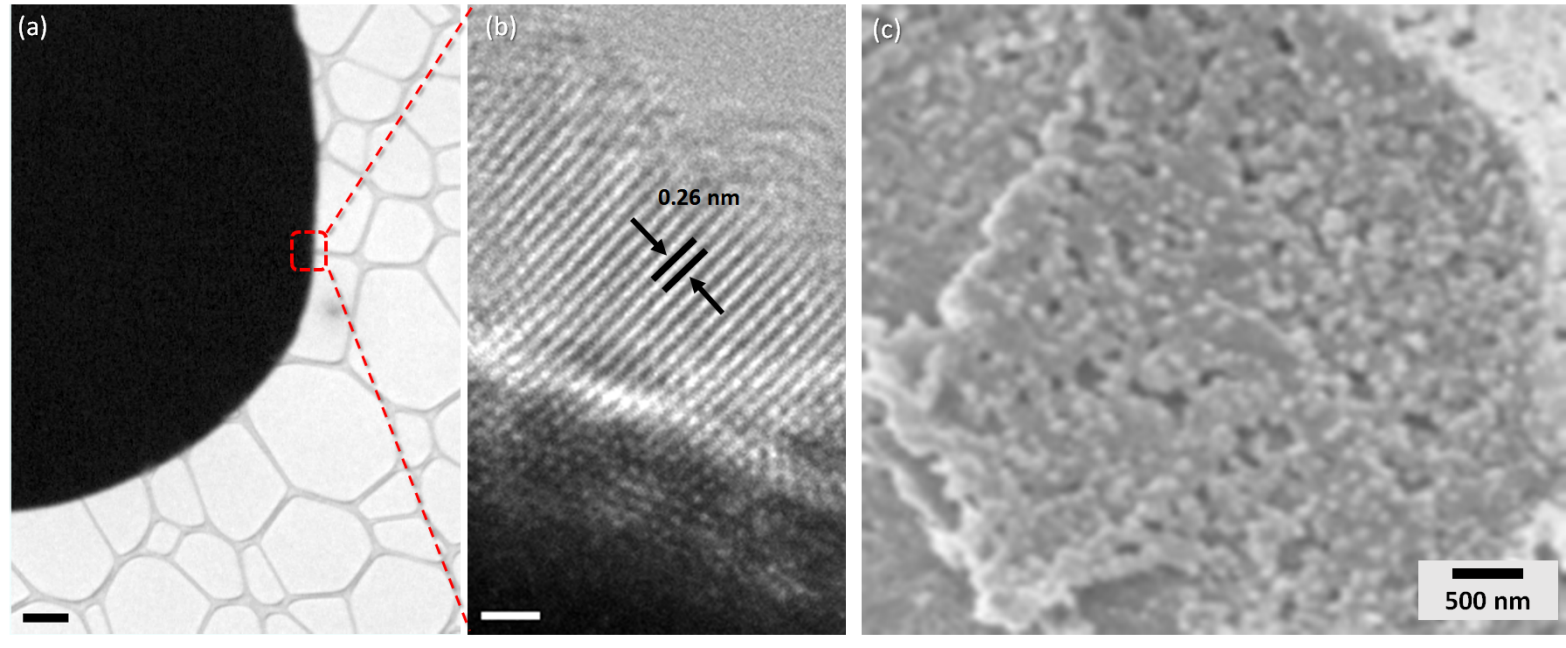
(**a**) Transmission electron microscopy (TEM) images of a single ZnO microsphere and (**b**) high magnification of the edge of the microsphere showing the lattice fringes. (**c**) High-magnification SEM images of a zinc oxide microsphere shell showing the porous nanosized zinc oxide particles. Scale bars: (**a**) 1 μm; and (**b**) 1 nm.

To further explore the crystal structure and morphology of the obtained ZnO microspheres, high-magnification SEM was conducted, as shown in Figure 4. Such a fine analysis gives a better view of the structure of the microcapsule shell and enables to give an approximate size of the ZnO nanoparticles building blocks, which is about 100 nm. These results strongly suggest that the formation mechanism of the microspheres is based on a non-classical crystallization pathway. In contrast to the classical crystallization, where the crystal is formed by clusters from building units such as atoms, ions, or molecules, the crystal growth mechanism behind the formation of our microspheres is in accordance with a particle-mediated process involving mesoscopic transformation of self-assembled, metastable or amorphous precursor particles inside the droplet. Subsequently, the primary zinc oxide nanoparticles formed in the early stages of the reaction reorganize within the droplet. Afterwards, the evaporation of the solvent gives rise to significant internal forces going from the inside to the outside of the droplet, thus promoting agglomeration of the aggregates on the contour of the microsphere.

TEM analysis was conducted to study the morphology of the ZnO nanoparticles building blocks. Figure 4a,b presents low and high magnification TEM images, respectively. The low magnification TEM image indicates that the ZnO microsphere has a rounded edges, which was previously observed by Liu et al. [36]. Further, the high magnification TEM image of an ultra-thin area of the microsphere’s edge points out that the nanoparticles are structurally uniform and well-oriented, as shown by the parallel lattice fringes. Besides, the corresponding TEM image exhibits a lattice spacing of 0.26 nm, which corresponds to the d-spacing of the (0001) planes [37].

### 3.2. Precise Control of Droplets Size and ZnO Microparticles

**Figure 5:**
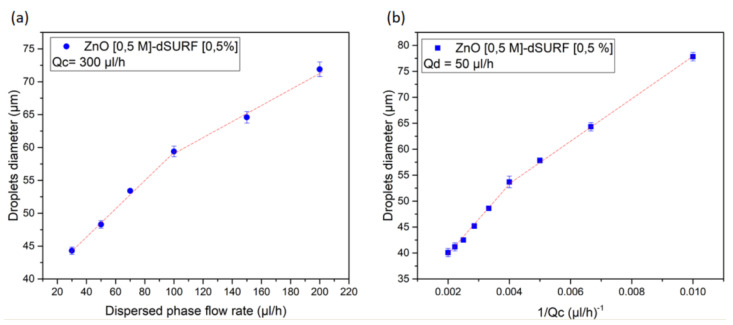
(**a**) Effect of the dispersed phase and (**b**) the continuous phase flow rates on the mean diameter of the generated droplets at a flow rate of the continuous and dispersed phases equal to $Q_{c}=300\;\mu L/h$ and $Q_{d}=50\;\mu L/h$, respectively.

The size of ZnO microparticles can be controlled precisely by adjusting the size of the initial droplets. Actually, the size of generated droplets depends on several parameters, such as the flow rates of the carrier (oil) phase and the dispersed phase and the geometry of the microfluidic device, as well as the interfacial tension and the viscosities of the two fluids. In the present study, we investigated the effect of flow rates of the dispersed phase and continuous phase on the droplet size, as illustrated in Figure 5a,b. Approximately 100 droplets were analyzed in each experiment. We observed that, for a given flow rate of the continuous phase equal to $Q_{c}$ = 300 μL/h, the size of droplets varies practically linearly with the flow rate of the dispersed phase ($Q_{d}$), from $Q_{d}$ = 30 μL/h to $Q_{d}$ = 100 μL/h and from $Q_{d}$ = 150 μL/h to $Q_{d}$ = 250 μL/h. Regardless of the break in the $Q_{d}$ = 100 μL/h to $Q_{d}$ = 150 μL/h range, the observed linear feature is actually consistent with a pinch-off mechanism of the droplet, a model which was initially developed by Garstecki et al. [38].

Indeed, the droplet size *L* varies with $Q_{d}$ according to the following relationship:

(2) $$\frac{L}{w}∼\tau \times Q_{d}$$

where *w* and τ represent the width of the channel, i.e., the nozzle, and the time to form a droplet, respectively. The time τ may be considered as the sum of the time $t_{fill}$ it takes the dispersed phase (sol) to fill the cross-section of the main channel and the time $t_{sq}$ to squeeze the emerging drop to detach it from the dispersed phase: $\tau =t_{fill}+t_{sq}$. Since $t_{fill}$ scales as $\frac{1}{Q_{d}}$ and $t_{sq}$ scales as $\frac{1}{Q_{c}}$, the (normalized) size of droplets should scale as:

(3) $$\frac{L}{w}∼1+\frac{Q_{d}}{Q_{c}}$$

The scaling law simplifies in the case where $Q_{d}>>Q_{c}$ to:

(4) $$\frac{L}{w}∼\frac{Q_{d}}{Q_{c}}$$

whereas, for $Q_{d}≃Q_{c}$, the droplets size becomes practically independent of $Q_{c}$ (and $Q_{d}$). These scaling laws are in very good agreement with our results, as shown in Figure 5a,b.

We attribute the observed break of the linear dependence of droplets size versus $Q_{d}$ flow rate, in the 100μL/h to 150μL/h range, as well as the observed difference in the dependence of pure and rhodamine doped ZnO microparticles versus the size of droplets, to the electrostatic forces between the dispersed charged ZnO nanoparticles inside and at the interface of the produced droplets in the microfluidic channel. The balance between such forces and other forces, i.e., interfacial forces, may depend on the size of droplets. This effect is discussed in detail in the following. It is well known that the (wurtzite) ZnO crystal lattice is constructed by alternating planes made of tetrahedrally coordinated Zn${}^{2+}$ and O${}^{2-}$ ions stacked along the c-axis direction. The structure of ZnO nanocrystals consequently consists of Zn${}^{2+}$ terminated (0001) face and O${}^{2-}$ terminated $\left(000\bar{1}\right)$ face, and ZnO nanocrystals possess an intrinsic dipole moment along their c-axis.

### 3.3. Effect of Electrical Charges on Droplets Stability and Microparticles Size

The addition of surfactants is generally mandatory in droplet-based microfluidic technology, as they enable for the stabilization of droplet interfaces and avoid uncontrolled merging of droplets when they come into contact [39,40]. In our study, since ZnO nanocrystals primary building blocks carry a net electric charge on their surfaces, one may expect that the electrical charge carried by surfactant molecules may play a crucial role in the stability of ZnO microdroplets. To check this hypothesis and understand how surfactants nature can affect the synthesis of ZnO droplets, we investigated the effect of a negatively charged fluoropolymer surfactant, which was obtained directly by adding a strong base, Benzyltrimethylammonium hydroxide (BTA), to the Krytox 157SH fluoropolymer (from 3M).

**Figure 6:**
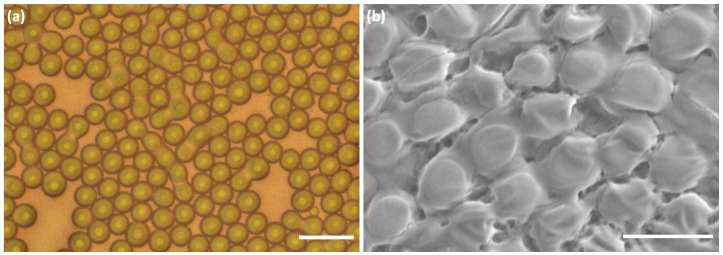
Optical (**a**) and SEM (**b**) micrographs of ZnO droplets stabilized by TB-Krytox surfactant. The observed merging process demonstrate the effect of screening charges of ZnO nanoparticles on the long term stability of zinc oxide droplets and particles. Scale bars: (**a**) 30 μm; and (**b**) 20 μm.

It can be observed in Figure 6 that droplets stabilized with the negatively charged surfactant (TB-Krytox) are stable only in the early stage of condensation. Droplets become unstable as they start to condense and their size starts to reduce significantly, unlike droplets stabilized by the non-charged surfactant (dSURF), as shown in Figure 2, which remain stable during the whole process of production and condensation of droplets. These observations demonstrate the crucial role of surface electric charges on the stability of ZnO microdroplets and microparticles and that ZnO microparticles carry in fact a net electric charge on their surfaces.

We suggest that the higher stability of ZnO microparticles in the presence of non-charged surfactant molecules is ensured by the repulsive electrostatic forces between charged ZnO nanoparticles, which aggregate at the droplets interface. In contrast, when using the charged BTA-Krytox surfactant, repulsive electrostatic forces should be screened by the negative charge of the terminal carboxylic acid of the BTA(+)-Krytox(−) surfactant monolayer.

### 3.4. Self-Organization and Orientation of ZnO Nanoparticles in the Microcapsules Shell

**Figure 7:**
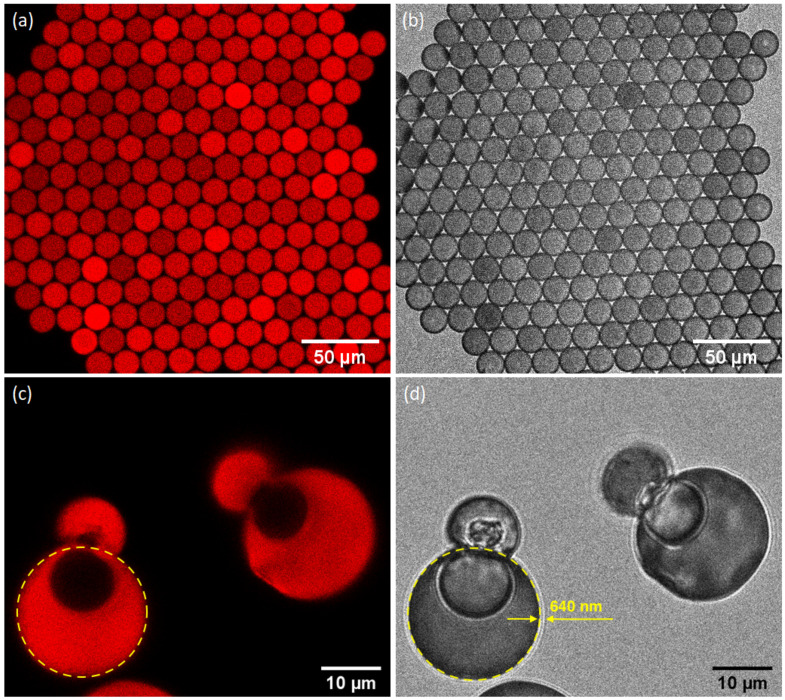
(**a**,**c**) Confocal fluorescence and (**b**,**d**) the corresponding white field microscopy images of 16.8 μm zinc oxide microspheres, (**c**,**d**) where we can see the top part of the microcapsule detached.

We show above the important effect of RhB molecules addition on the relationship between the size of ZnO microparticles and the size of the initial droplets. As often observed during oriented aggregation-mediated growth of primary nanoparticles in the presence of soluble organic molecules, such as polymers and surfactants [41,42], RhB molecules would be expected to serve as intercalated bridges between aggregated ZnO nanoparticles during the aggregation process. To better understand this effect and to give a further insight into the aggregation process of primary ZnO nanoparticles leading to the formation of ZnO microcapsules, we doped ZnO nanoparticles dispersion with a solution of rhodamine B, at a concentration $C_{B}=0.1\;$mM, and used confocal microscopy to analyze the fluorescence and structural properties of the as-synthesized dye-doped microcapsules, as shown in Figure 7. White-field image of this figure shows that ZnO microspheres are in close contact with each other. In contrast, the corresponding fluorescence image reveals that only the inner part of the microcapsules is fluorescent, i.e., RhB dye is excluded from the shell of the microcapsules.

It is noteworthy that microparticles, when imaged using confocal microscopy, are re-suspended in HFE oil, in order to facilitate their homogeneous transfer from the Petri dish, where they have been formed, onto the microscope plate. Therefore, and because of their demonstrated porosity, microcapsules absorb oil, swell, and eventually their shell breaks, as shown in Figure 7c,d. This is also the reason their size appears larger by regards to the deduced size from SEM analysis, shown in Figure 2 (where microcapsules are fully dry).

In addition, because of the presence of oil inside microcapsules, they appear, in Figure 7a, as a full-like structure and not as hollow microspheres. Indeed, during the swelling process of the microcapsules, RhB molecules which are adsorbed on the inner surface of the microcapsule shell are transported by the incoming oil flux and thus the confined oil inside the microcapsules becomes fluorescent and microcapsules appear as full fluorescent microspheres. In the case of open microcapsules, the fluorescent oil is expelled out of the microcapsule and subsequently their interior appears dark, as shown in Figure 7c. This result confirms again the hollow structure of the microcapsules.

**Figure 8:**
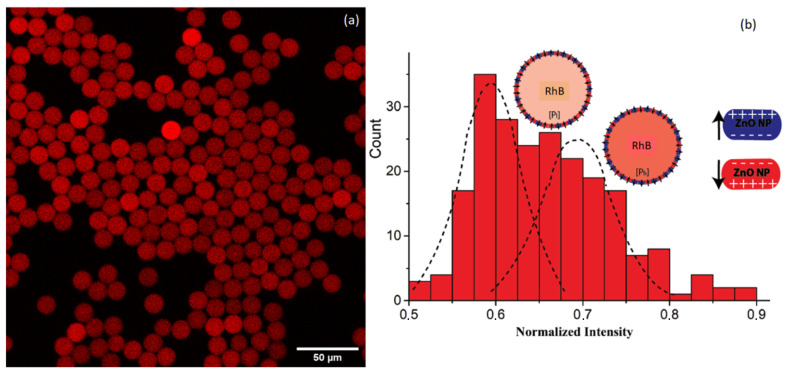
(**a**) Confocal fluorescence showing a non homogeneous distribution of rhodamine B dye in different ZnO microcapsules and (**b**) the corresponding distribution of normalized fluorescence intensity showing two populations of microcapsules.

Careful analysis of fluorescence confocal images also gives a valuable information on the organization of ZnO nanoparticles and the distribution of the microcapsules surface electrical charges. We show in Figure 8a a confocal fluorescence microscopy image of another sample of rhodamine B-doped ZnO microcapsules. They were obtained from droplets with a typical size of 40 μm, where we can see a noticeable discrepancy in the fluorescence intensity of the different microcapsules.

The distribution of the normalized fluorescence intensity of the different microcapsules is shown in Figure 8b (approximately 222 microcapsules). We can see clearly two types of microcapsules, one type that we call $P_{l}$ (where “*l*” stands for “low intensity”), with a normalized fluorescence intensity centered around 0.6 and a second type that we name $P_{h}$ (where “*h*” stands for “high intensity”), with a fluorescence intensity centered around 0.7.

To better understand the observed distribution of the fluorescence intensity and the coexistence of two populations of microcapsules, $P_{l}$ and $P_{h}$, it is important to keep in mind that all droplets and microcapsules contain initially the same quantity of rhodamine B dye, [RhB]=0.1 mm, and that microcapsules are full with HFE oil. Therefore, we should attribute such result to a difference in the final concentration of RhB molecules in the oil, which is confined microcapsules.

We argue in the following that the observed fluorescence intensity distribution should be related to a difference in the interaction between rhodamine B molecules and the internal surface of the microcapsule shell, which may vary from one microcapsule to another. Indeed, rhodamine B molecules may undergo strong electrical interaction with the positively charged surface Zn${}^{2+}$ planes of ZnO nanoparticles because of their negative electrical charge carried by acetate groups. In addition, at the used basic pH = 8.2, rhodamine B molecules exhibit a zwitterionic form, and hence may interact also with the negatively charged O${}^{2-}$ planes of ZnO nanoparticles through the positive charge carried by their ammonium group. However, these two types of interactions may not have the same strength. From a chemical point of view, zinc acetate complexes are known to be stable chemical species. We should note that zinc acetate was initially used as a precursor for the synthesis of ZnO nanoparticles.

Consequently, we suggest that depending on the orientation of ZnO nanoparticles at the inner surface of the shell of the microcapsules, RhB molecules would adsorb more or less strongly on a such surface. The higher is the density of nanoparticles with their Zn${}^{2+}$ planes oriented inward, the higher is the number of adsorbed rhodamine B molecules on the inner surface of the shell and the lower is the number of rhodamine B molecules which are dissolved and transported by the inward flux of HFE oil while filling the microcapsules.

As shown in Figure 8b, we observed two types of microcapsules, one with a higher fluorescence intensity and another one with a lower one. This result may suggest that two types of ZnO nanoparticles orientations coexist at the inner surface of the microcapsules with distributions roughly in equal proportions.

Therefore, we may conclude that approximately 50% of microcapsules point their positively charged Zn${}^{2+}$ planes towards the inner part of the shell and hence 50% of microcapsules orient their negatively charged O${}^{2-}$ planes towards the inner part of the shell, as sketched in Figure 8b.

### 3.5. Dynamics of Rhodamine Dye Molecules and Electrical Charges Distribution on the Surface of the Microcapsule Shell

**Figure 9:**
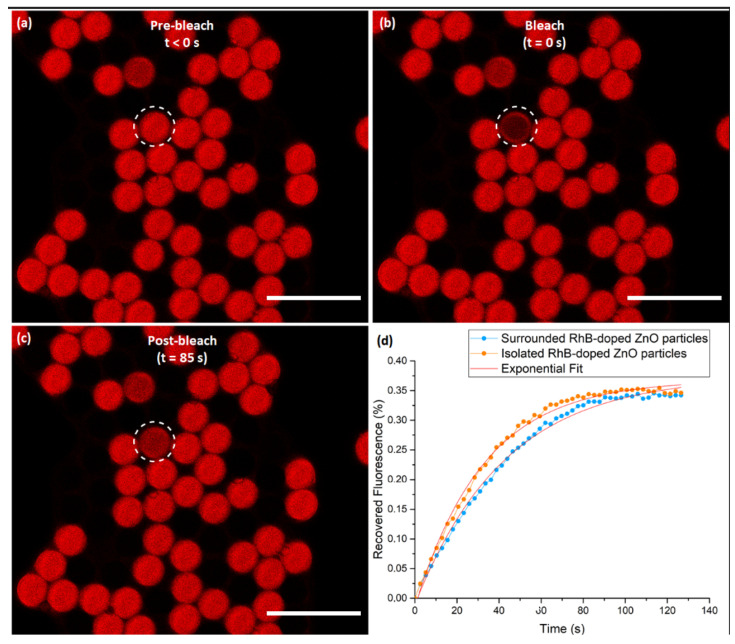
Confocal microscopy images recorded during a FRAP experiment on ZnO microspheres uniformly labeled with rhodamine B dye: (**a**) taken before photobleaching; (**b**) immediately after photobleaching the spherical inner part of the microsphere, with short pulse of intense laser light, showing that the selective ROI is no longer fluorescent; (**c**) recorded during the recovery after photobleaching; and (**d**) the recovery curve that demonstrates an increase in fluorescence intensity as the bleached dye diffuses and the new unbleached dye move into the ROI. The white dashed circles represent the selected microsphere for photobleaching. Scale bars: 50 μm.

To establish a quantitative analysis on the dynamics of RhB molecules mobility within and across the microcapsules, or between other neighbouring microcapsules, we performed fluorescence recovery after photobleaching (FRAP) experiments. These experiments were carried out in target ROI (region of interest) of microcapsules with 16.5 μm diameter and doped with rhodamine B dye. The size of ROI was constant for all measures, that is A(ROI)=113 μm${}^{2}$. The concentration of the dye, [RhB]=0.1 mm, has been chosen small enough so that RhB molecules should be rapidly and permanently photobleached when illuminated with the laser beam. Practically, we uniformly photobleached the dye located on the inner part of two types of microspheres: those that are surrounded by other microcapsules (as shown in Figure 9a–c) and those that are isolated. Next, we plotted fluorescence intensity of the ROI after photobleaching as a function of time, as shown in Figure 9d, where blue and orange graphs illustrate the average fluorescence intensity recovered for surrounded microcapsules and isolated microcapsules, respectively.

It is important to note that the recovered fluorescence intensity exhibits the same dynamics for both types of microcapsules (isolated and surrounded). We found in particular a similar value for the final recovered intensity, that is 35% of the initial fluorescence intensity (measure in the same ROI before photobleaching) (which is reached after a period of time of about 1 min). These results provide very interesting evidence that the diffusion of RhB molecules inside microcapsules does not dependent on the environment of the studied microcapsules. It is important to note that in this case RhB molecules cannot pass the microcapsule shell barrier and are consequently well confined within the microcapsule. Consequently, rhodamine B molecules remain attached to the microsphere, and thus there is no exchange between the microsphere and its neighboring molecules after the photobleaching. This result is also in a good agreement with fluorescence confocal images, which shows clearly that RhB molecules stay confined on the inner surface of the shell and do not diffuse inside the shell.

The diffusion coefficient $D_{RhB}$ of RhB dye molecules inside the microcapsules (filled with HFE oil) may be deduced directly from the measure of the half-time $t_{1/2}$ of fluorescence recovery, according to the following equation:

$$D_{RhB}=\frac{A(ROI)}{t_{1/2}}$$

From the graphs shown in Figure 9d, we deduce a value $t_{1/2}≃$ 30 s and hence a mean diffusion constant value for RhB molecules inside the microcapsules of about $D_{RhB}=$ 3.8 $\times 10^{-8}$ cm${}^{2}$/s.

Moreover, as the measured diffusion coefficient value is found to be smaller by two order of magnitude in regards to the literature value of rhodamine B in water (e.g., $D=3.6\times 10^{-6}{\mathrm{cm}}^{2}/s$ [43]), we may then conclude that rhodamine B molecules should be strongly attracted to the inner surface of ZnO microcapsules because of strong electrostatic interactions between electrically charged RhB molecules and the electrical charges carried by ZnO nanoparticles.

We suggest that, similar to the observed discrepancy in the fluorescence intensity of different microcapsules shown in Figure 8, we also expect a discrepancy in the measured values of diffusion coefficient, as the overall electrical charge carried by the inner surface of the shell may vary from one microcapsule to another depending on the effective orientation of the different ZnO nanoparticles building blocks. A more detailed study will be carried in the future in order to investigate in detail this effect.

## 4. Conclusions and Perspectives

In this paper, we show that droplet-based microfluidics enables the synthesis of tunable and stable ZnO microspheres. The as-prepared ZnO mesocrystal microspheres form through a non-classical crystallization mechanism from the building blocks of ZnO nanoparticles. Further, our experiments and theoretical calculations confirmed the development of ZnO microspheres with hollow structure, i.e., microcapsules. We investigated the effect of different parameters, such as the effect of flow rates of the dispersed and the continuous phases. We also investigated the structure of the shell of the microspheres at the microscale using mainly scanning electron microscopy and confocal microscopy in the presence of a charged dye (rhodamine B) and used transmission electron microscopy to have a better insight into the organization of the primary ZnO nanocrystals building blocks of the microcapsules. We showed in this study that the measurement of the diffusion coefficient of dye molecules within ZnO microcapsules could be easily performed using FRAP experiments. In this context, our results confirms the strong interaction between dye molecules and the electrical charges carried by ZnO nanoparticles. Our results demonstrate that droplet microfluidics provides an efficient and promising approach not only for the fabrication of highly monodisperse ZnO microcapsules with a tunable size and a precise control at the microscale level but also for better understanding of colloidal growth processes and interaction with dye molecules. Besides, our approach enables for the production of well-defined monodisperse ZnO microcapsules with a high control of size and chemical composition according to the use of highly monodisperse droplets as soft-templates for their synthesis. The as-synthesized ZnO microcapsules may find interesting applications in drug delivery, biosensing, photonic, and energy applications.
